# Supplementary material for: HDAC6 inhibition as a mechanism to prevent neurodegeneration in the mSOD1G93A mouse model of ALS
Source: Heliyon. 2024 Jul 14;10(14):e34587. doi: 10.1016/j.heliyon.2024.e34587 (PMC11315133; doi:10.1016/j.heliyon.2024.e34587)
Supplement: Multimedia component 1 [file mmc1.docx]

**Supplementary Table 1:**Serum NFL levels are unaltered in mSOD1^G93A^ mice treated with ACY-738

| *Predictors* | *Sex* | *N* | *Age (weeks)* | *Mean log NFL(pg/uL)* | *95%CI* |
| --- | --- | --- | --- | --- | --- |
| mSOD1^G93A^ vehicle | F | 8 | 14 | 6.81 | 6.50 – 7.13 |
| mSOD1^G93A^ vehicle | M | 9 | 14 | 7.11 | 6.80 – 7.42 |
| mSOD1^G93A^riluzole | F | 8 | 14 | 6.77 | 6.46 – 7.09 |
| mSOD1^G93A^riluzole | M | 8 | 14 | 7.04 | 6.75 – 7.34 |
| mSOD1^G93A^ ACY-738 | F | 9 | 14 | 6.52 | 6.19 – 6.85 |
| mSOD1^G93A^ ACY-738 | M | 8 | 14 | 7.01 | 6.68 – 7.35 |
| mSOD1^G93A^ ACY-738 + riluzole | F | 10 | 14 | 6.76 | 6.46 – 7.07 |
| mSOD1^G93A^ ACY-738 + riluzole | M | 8 | 14 | 6.91 | 6.61 – 7.21 |
| mSOD1^G93A^ vehicle | F | 8 | 20 | 8.41 | 8.12 – 8.70 |
| mSOD1^G93A^ vehicle | M | 9 | 20 | 8.17 | 7.88 – 8.47 |
| mSOD1^G93A^riluzole | F | 8 | 20 | 8.04 | 7.74 – 8.34 |
| mSOD1^G93A^riluzole | M | 8 | 20 | 8.36 | 8.06 – 8.66 |
| mSOD1^G93A^ ACY-738 | F | 9 | 20 | 7.89 | 7.61 – 8.18 |
| mSOD1^G93A^ ACY-738 | M | 8 | 20 | 8.16 | 7.87 – 8.46 |
| mSOD1^G93A^ ACY-738 + riluzole | F | 10 | 20 | 8.17 | 7.91 – 8.45 |
| mSOD1^G93A^ ACY-738 + riluzole | M | 8 | 20 | 8.01 | 7.71 – 8.31 |

Data presented as conditional log(mean) and 95% confidence intervals.

**Supplementary Table 2:**All paw grip strength comparison between mSOD1^G93A^ andC57BL/6 control mice*.*

| *Predictors* | *Sex* | *N* | *mean* | *95%CI* |
| --- | --- | --- | --- | --- |
| C57BL/6 | F | 5 | 211 | 191 – 231 |
| C57BL/6 | M | 5 | 216 | 188 - 245 |
| mSOD1^G93A^ vehicle | F | 16 | 118 | 101-136 |
| mSOD1^G93A^ vehicle | M | 15 | 130 | 112-148 |
| mSOD1^G93A^riluzole | F | 12 | 129 | 109-149 |
| mSOD1^G93A^riluzole | M | 11 | 141 | 120-162 |
| mSOD1^G93A^ ACY-738 | F | 18 | 117 | 101-134 |
| mSOD1^G93A^ ACY-738 | M | 15 | 124 | 107-142 |
| mSOD1^G93A^ ACY-738 + riluzole | F | 20 | 123 | 108 - 139 |
| mSOD1^G93A^ ACY-738 + riluzole | M | 11 | 135 | 114 - 155 |

Data presented as conditional means and 95% confidence intervals.

**Supplementary Table 3:**Latency to fall from wire-hang task is not altered after treatment with ACY-738*.*

| *Predictors* | *Sex* | *N* | *Mean (s)* | *95%CI* |
| --- | --- | --- | --- | --- |
| WT | F | 5 | 136.7 | 103.1 – 170.3 |
| WT | M | 5 | 157.7 | 120.8 – 194.5 |
| mSOD1^G93A^ vehicle | F | 16 | 3.67 | 3.32 - 4.01 |
| mSOD1^G93A^ vehicle | M | 15 | 3.68 | 3.33 – 4.04 |
| mSOD1^G93A^riluzole | F | 14 | 3.93 | 3.49 - 4.37 |
| mSOD1^G93A^riluzole | M | 11 | 3.07 | 2.59 - 3.54 |
| mSOD1^G93A^ ACY-738 | F | 18 | 3.81 | 3.44 – 4.18 |
| mSOD1^G93A^ ACY-738 | M | 15 | 3.02 | 2.59 - 3.54 |
| mSOD1^G93A^ ACY-738 + riluzole | F | 19 | 3.85 | 3.49 – 4.21 |
| mSOD1^G93A^ ACY-738 + riluzole | M | 11 | 2.98 | 2.50 – 3.46 |

Data presented as conditional means and 95% confidence intervals.

**Supplementary Table 4:**Neuromuscular tremor onset in mSOD1^G93A^ mice treated with ACY-738.

| *Predictors* | *Sex* | *N* | *Mean (week of onset)* | *95%CI* |
| --- | --- | --- | --- | --- |
| mSOD1^G93A^ vehicle | F | 16 | 15.6 | 14.8 – 16.5 |
| mSOD1^G93A^ vehicle | M | 14 | 15.0 | 14.1 – 16.0 |
| mSOD1^G93A^riluzole | F | 12 | 15.7 | 14.7 – 16.8 |
| mSOD1^G93A^riluzole | M | 11 | 15.1 | 14.1 – 16.2 |
| mSOD1^G93A^ ACY-738 | F | 18 | 14.7 | 13.9 – 15.6 |
| mSOD1^G93A^ ACY-738 | M | 15 | 13.9 | 13.0 – 14.9 |
| mSOD1^G93A^ ACY-738 + riluzole | F | 20 | 14.9 | 14.1 – 15.7 |
| mSOD1^G93A^ ACY-738 + riluzole | M | 11 | 14.1 | 13.1 – 15.1 |

Data presented as conditional means and 95% confidence intervals.

**Supplementary Table 5:**SMI312 positive axons are rescued by riluzole treatment in mSOD1^G93A^ mice.

| *Predictors* | *Sex* | *N* | *mean* | *95%CI* |
| --- | --- | --- | --- | --- |
| C57BL/6 WT | F | 4 | 4.35 | 3.83 – 4.88 |
| C57BL/6 WT | M | 4 | 4.24 | 3.72 – 4.77 |
| mSOD1^G93A^ vehicle | F | 9 | 3.39 | 3.06 – 3.73 |
| mSOD1^G93A^ vehicle | M | 7 | 3.21 | 2.86 – 3.55 |
| mSOD1^G93A^riluzole | F | 8 | 3.82 | 3.47 – 4.16 |
| mSOD1^G93A^riluzole | M | 7 | 3.63 | 3.28 – 3.98 |
| mSOD1^G93A^ ACY-738 | F | 6 | 3.64 | 3.26 – 4.01 |
| mSOD1^G93A^ ACY-738 | M | 8 | 3.45 | 3.09 – 3.82 |
| mSOD1^G93A^ ACY-738 + riluzole | F | 14 | 3.83 | 3.54 – 4.12 |
| mSOD1^G93A^ ACY-738 + riluzole | M | 7 | 3.65 | 3.31 – 3.98 |

Data presented as conditional means and 95% confidence intervals.

**Supplementary Table 6:**ChAT positive axons are rescued by ACY-738 and riluzole treatment in female mSOD1^G93A^ mice.

| *Predictors* | *Sex* | *N* | *mean* | *95%CI* |
| --- | --- | --- | --- | --- |
| C57BL/6 WT | F | 4 | 2.86 | 2.41 – 3.32 |
| C57BL/6 WT | M | 4 | 2.88 | 2.43 – 3.34 |
| mSOD1^G93A^ vehicle | F | 9 | 2.07 | 1.83 – 2.30 |
| mSOD1^G93A^ vehicle | M | 7 | 2.37 | 2.11 – 2.62 |
| mSOD1^G93A^riluzole | F | 8 | 2.17 | 1.92 – 2.41 |
| mSOD1^G93A^riluzole | M | 7 | 2.10 | 1.84 – 2.36 |
| mSOD1^G93A^ ACY-738 | F | 6 | 2.12 | 1.83 – 2.40 |
| mSOD1^G93A^ ACY-738 | M | 8 | 2.08 | 1.82 – 2.34 |
| mSOD1^G93A^ ACY-738 + riluzole | F | 14 | 2.47 | 2.29 – 2.66 |
| mSOD1^G93A^ ACY-738 + riluzole | M | 7 | 2.28 | 2.02 – 2.54 |

Data presented as conditional means and 95% confidence intervals.

**Supplementary Table 7:**Lumbar motor neuron counts in mSOD1^G93A^ mice treated with ACY-738.

| *Predictors* | *Sex* | *N* | *mean* | *95%CI* |
| --- | --- | --- | --- | --- |
| C57BL/6 WT | F | 4 | 11.33 | 10.31 – 12.34 |
| C57BL/6 WT | M | 4 | 11.90 | 10.88 – 12.93 |
| mSOD1^G93A^ vehicle | F | 8 | 6.66 | 5.98 – 7.33 |
| mSOD1^G93A^ vehicle | M | 6 | 7.86 | 7.12 – 8.61 |
| mSOD1^G93A^riluzole | F | 9 | 8.07 | 7.45 – 8.68 |
| mSOD1^G93A^riluzole | M | 6 | 7.63 | 6.86 – 8.40 |
| mSOD1^G93A^ ACY-738 | F | 8 | 8.41 | 7.74 – 9.08 |
| mSOD1^G93A^ ACY-738 | M | 6 | 7.69 | 6.90 – 8.47 |
| mSOD1^G93A^ ACY-738 + riluzole | F | 9 | 7.23 | 6.59 – 7.86 |
| mSOD1^G93A^ ACY-738 + riluzole | M | 6 | 7.71 | 6.93 – 8.49 |

Data presented as conditional means and 95% confidence intervals.

**Supplementary Table 8:**Iba1 positive microglia are unaltered after treatment with ACY-738.

| *Predictors* | *Sex* | *N* | *mean* | *95%CI* |
| --- | --- | --- | --- | --- |
| C57BL/6 WT | F | 4 | 3.75 | -0.07 – 7.58 |
| C57BL/6 WT | M | 4 | 2.26 | -1.57 – 6.09 |
| mSOD1^G93A^ vehicle | F | 11 | 12.18 | 9.60 – 15.0 |
| mSOD1^G93A^ vehicle | M | 10 | 11.75 | 7.99 – 13.6 |
| mSOD1^G93A^riluzole | F | 8 | 13.45 | 8.53 – 18.4 |
| mSOD1^G93A^riluzole | M | 7 | 13.23 | 7.98 – 18.5 |
| mSOD1^G93A^ ACY-738 | F | 11 | 12.10 | 7.90 – 16.3 |
| mSOD1^G93A^ ACY-738 | M | 10 | 10.26 | 5.87 – 14.7 |
| mSOD1^G93A^ ACY-738 + riluzole | F | 16 | 9.04 | 5.57 – 12.5 |
| mSOD1^G93A^ ACY-738 + riluzole | M | 8 | 9.62 | 4.69 – 14.6 |

Data presented as conditional means and 95% confidence intervals.

**Supplementary Table 9:**Astrocyte reactivity is unaltered in mSOD1^G93A^ mice treated with ACY-738

| *Predictors* | *Sex* | *N* | *mean* | *95%CI* |
| --- | --- | --- | --- | --- |
| C57BL/6 WT | F | 4 | 1.92 | -1.55 – 5.38 |
| C57BL/6 WT | M | 4 | 0.44 | -3.04 – 3.92 |
| mSOD1^G93A^ vehicle | F | 11 | 12.72 | 9.70 – 15.7 |
| mSOD1^G93A^ vehicle | M | 7 | 10.30 | 6.56 – 14.0 |
| mSOD1^G93A^riluzole | F | 8 | 9.07 | 5.55 – 12.6 |
| mSOD1^G93A^riluzole | M | 7 | 11.74 | 8.01 – 15.5 |
| mSOD1^G93A^ ACY-738 | F | 9 | 16.44 | 13.12 – 19.8 |
| mSOD1^G93A^ ACY-738 | M | 10 | 12.84 | 9.71 – 16.0 |
| mSOD1^G93A^ ACY-738 + riluzole | F | 14 | 12.08 | 9.45 – 14.7 |
| mSOD1^G93A^ ACY-738 + riluzole | M | 8 | 9.07 | 6.01 – 13.0 |

Data presented as conditional means and 95% confidence intervals.


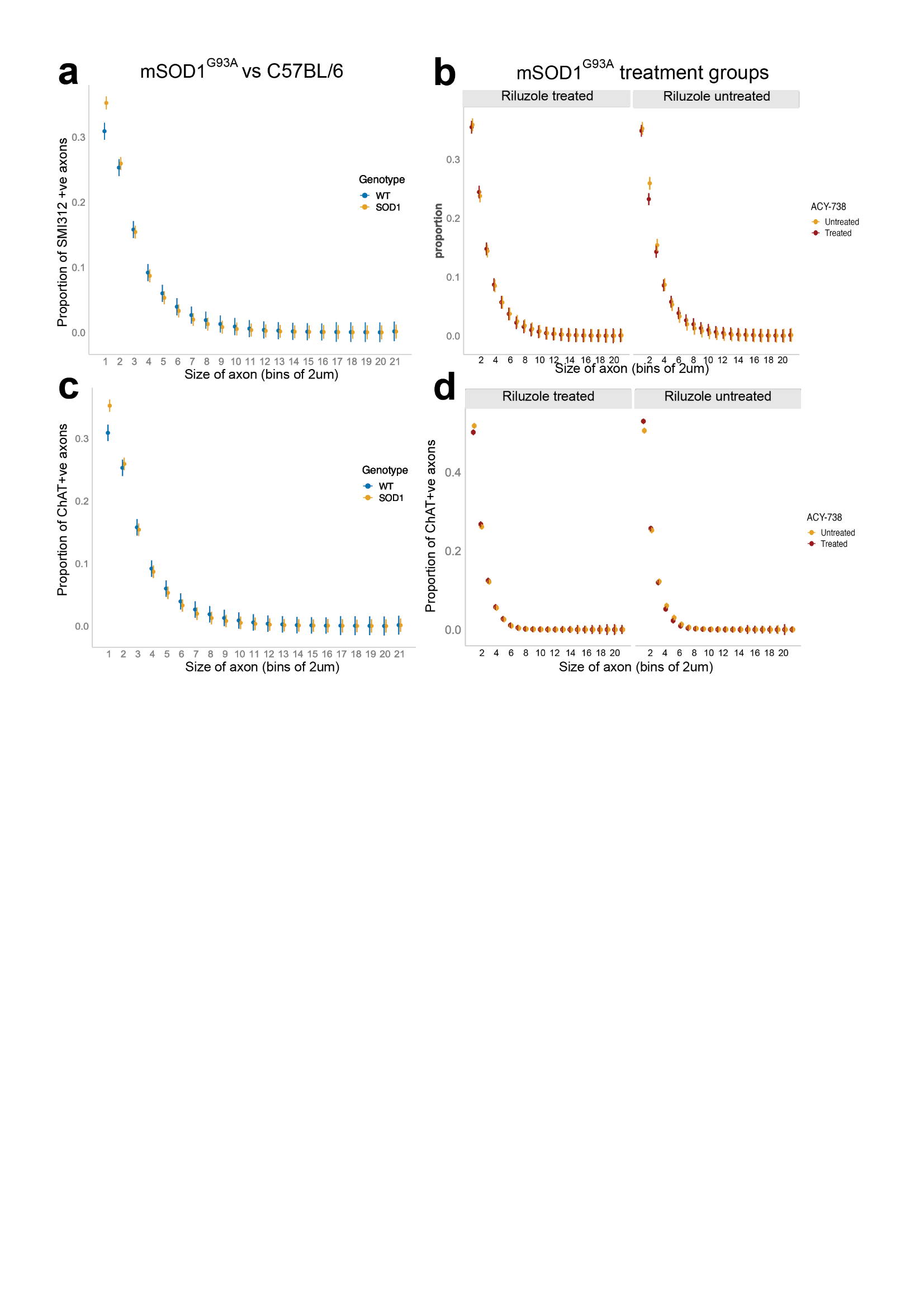


***Supplementary Figure 1:Riluzole treatment reduces the proportion of small axons in the sciatic nerve of mSOD1^G93A^ mice***

Plots showing the proportion of SMI-312 labelled axons (a-b) and ChAT (c-d) labelled axons in the sciatic nerve of C57BL/6 and mSOD1^G93A^ mice (a,c), and mSOD1^G93A^ treatedmice (b,d). Axons are grouped into 2µm bins across the x-axis. There was a significant increase in the proportion of small (2-6µm bins) SMI-312 labelled axons after treatment with riluzole. BothACY-738 and riluzole treatment significantly increased the proportion of small (0.4-2µm)ChAT-labelled axons in ^mSOD1G93A^ mice.
